# Longitudinal changes in endocannabinoids and clinical course of adolescent non-suicidal self-injury

**DOI:** 10.1038/s41398-026-04391-x

**Published:** 2026-09-03

**Authors:** Marc D. Ferger, Susanne Brodesser, Michael Kaess, Julian Koenig

**Affiliations:** 1 University of Cologne, Faculty of Medicine and University Hospital Cologne, Department of Child and Adolescent Psychiatry, Psychosomatics and Psychotherapy, Cologne, Germany; 2 University Hospital Heidelberg, Centre for Psychosocial Medicine, Department of Child and Adolescent Psychiatry, Heidelberg, Germany; 3 University of Ulm, Department for Child and Adolescent Psychiatry/Psychosomatics/Psychotherapy, Ulm, Germany; 4 University of Calgary, Department of Psychiatry, Mathison Centre for Mental Health Research and Education, Calgary, Canada; 5 University of Cologne, Faculty of Medicine and University Hospital of Cologne, Cluster of Excellence on Cellular Stress Responses in Aging-Associated Diseases (CECAD), Cologne, Germany; 6 University of Bern, University Hospital of Child and Adolescent Psychiatry and Psychotherapy, Bern, Switzerland

**Keywords:** Prognostic markers, Physiology

## Abstract

Non-suicidal self-injury (NSSI) in adolescents has previously been linked to alterations in the endocannabinoid system. While increasing evidence supports endocannabinoid levels as potential biomarker in psychiatric care, little is known about their longitudinal trajectories over the course of treatment or in predicting clinical symptoms. This is the first study to investigate endocannabinoids in a longitudinal cohort of patients with NSSI, exploring associations with the development of symptoms over time. Plasma endocannabinoids and NSSI, using the German version of the *Self-Injurious Thoughts and Behaviors Interview* (SITBI-G), were assessed in *n* = 47 female adolescents with NSSI at both baseline and after one year of treatment (follow-up). Associations between endocannabinoid levels and clinical symptoms over time were analyzed. Anandamide (AEA) significantly decreased from baseline to follow-up while 2-arachidonoylglycerol (2-AG) increased after one year. While lower AEA levels have previously been reported in adolescents with NSSI, this longitudinal study found that AEA decreased over one year despite an overall improvement in NSSI symptoms. The overall pattern suggests that changes in circulating endocannabinoids do not closely correspond to symptom improvement. These findings indicate that circulating endocannabinoid levels change over time in adolescents with NSSI, but their complex and partly inconsistent associations with clinical symptoms limit their clinical utility as biomarkers.

## Introduction

Non-suicidal self-injury (NSSI) is defined as the deliberate, self-directed damage of own body tissue without suicidal intent, for purposes not socially or culturally sanctioned [1]. In the DSM-5, NSSI has been proposed as a discrete diagnostic entity, when the behavior is characterized by self-injurious episodes on five or more days in the past year. NSSI typically peaks between 14 and 15 years of age and declines into adulthood [2, 3]. It is more prevalent in females [4, 5] and has been observed in several psychiatric conditions most notably depression, posttraumatic stress disorder (PTSD), and borderline personality disorder (BPD) [6–9]. Importantly, NSSI is a strong predictor of future suicide attempts [10]. NSSI is commonly observed in patients with a history of childhood trauma and several studies have suggested a potential role of traumatic experiences in its development [11]. It is widely accepted that NSSI presents a (dysfunctional) coping strategy to regulate distressing emotional states. Psychotherapy (i.e., dialectical behavior therapy for adolescents (DBT-A) and cognitive behavioral therapy (CBT)), aiming at reducing dysfunctional and promoting functional coping strategies, are effective in the treatment of adolescent NSSI [12–14]. Neurobiologically informed interventions (i.e., pharmacology or neurostimulation) are currently not available for patients with NSSI [15, 16].

The endocannabinoid system is a neuromodulatory system and key player in the pathophysiology of stress, anxiety and fear [17–19]. The major molecular target of the endocannabinoid receptor is the CB1-receptor, the most highly expressed G protein-coupled receptor in the brain [20, 21]. The CB1-receptor is targeted by two major endogenous ligands, the endocannabinoids N-arachidonyl ethanolamide (anandamide, AEA) and 2-archidonyl glycerol (2-AG) [22]. The endocannabinoid system plays an important role in the stress response. Acute stress typically results in a transient reduction in brain AEA in the amygdala, prefrontal cortex (PFC), hippocampus and hypothalamus (Hill et al., [22]; Lutz et al., [17]; Morena et al., [23]) which facilitates rapid adaptation to stress and plays a role in regulating stress and anxiety [24]. Threat conditioning in humans has been shown to decrease AEA levels, with this reduction correlating with heightened amygdala activity during threat learning [25]. This effect appears to be mediated by AEA’s facilitation of excitatory activity in principal neurons through the disinhibition of parvalbumin-positive interneurons in the lateral amygdala [26]. A decrease in AEA allows for the stress-induced activation of the hypothalamic-pituitary-adrenal (HPA) axis, as AEA is thought to act as a tonic “gatekeeping” signal towards the HPA axis. [23, 27]. Chronic stress has been shown to stimulate fatty acid amide hydrolase (FAAH) activity via corticotropin-releasing hormone (CRH), ultimately resulting in a reduction in AEA concentration [28, 29]. Closing the feedback loop, animal research demonstrated, that glucocorticoids increase endocannabinoids [30] and mobilize the second main endocannabinoid 2-AG to suppress the excitatory inputs stemming from stress exposure and ultimately enable basal levels of activity in stress response systems [23].

In general, a wide body of research has demonstrated, that the endocannabinoid system plays a pivotal role in modulating other neuroendocrine systems like the HPA-axis, and is similarly implicated by those systems, often via feedback loops [31]. This presents a significant challenge in interpreting the role of these interactions in complex psychiatric conditions, where multiple systems may be dysregulated. Unsurprisingly, findings on endocannabinoids in psychiatric disorders are heterogeneous, with studies reporting both increased and decreased endocannabinoid levels across various conditions [32–34]. Recently, we demonstrated alterations of the endocannabinoid system in a large sample of adolescents with NSSI [35]: We showed that AEA was lower in adolescents with NSSI compared to typically developing controls, and AEA was negatively correlated with NSSI frequency and the severity of childhood trauma.

The present study aimed to add to these findings, by investigating the longitudinal development of plasma endocannabinoid levels in adolescents with NSSI, clarifying their potential role in symptom development and treatment response. We hypothesized that NSSI symptoms and endocannabinoid levels would change over one year. Given our cross-sectional finding of reduced AEA in adolescents with NSSI, we expected symptom improvement to be accompanied by a corresponding increase in AEA, reflecting a potential normalization of endocannabinoid tone alongside clinical recovery.

## Materials and methods

### Participants

Patients with NSSI were recruited from the specialized outpatient clinic for risk-taking and self-harm behavior “*Ambulanz für Risikoverhalten und Selbstschädigung (AtR!Sk)*” [36] at the Department of Child and Adolescent Psychiatry, University Hospital Heidelberg. Following an initial diagnostic assessment, eligible patients were invited within six weeks to join the AtR!Sk-Bio cohort, a longitudinal study focused on identifying biological correlates of risk-taking and self-harm behaviors in adolescence. The initial AtR!Sk-Bio cohort assessment is referred to as “baseline” in this manuscript and is described in detail later. After one year, participants were invited to undergo the same diagnostic procedures, referred to as “follow-up”.

Eligibility criteria included an age range of 12–17 years, with patients reporting at least five episodes of NSSI in the past year, as defined by DSM-5 criteria [37]. Patients with acute psychotic symptoms were excluded; however, given the high comorbidity with other psychiatric disorders, co-occurring diagnoses were permitted (see Table 1). Additional exclusion criteria included pregnancy, primary neurological, endocrinological, or cardiovascular disorders, and insufficient speech comprehension. Furthermore, due to sex differences in NSSI prevalence and endocannabinoid signaling [38, 39], only female participants were included in the current analyses. The Ethics Committee of the Faculty of Medicine, University of Heidelberg, approved the scientific evaluation of AtR!Sk (IRB approval number S-449/2013) and the additional neurobiological assessments (IRB approval number S-514/2015). All participants and their caregivers provided written informed consent and received an allowance of €40 for their participation.

**Table 1:** Sociodemographic characteristics of the study sample.

Variable	Group; mean ± SD or *N* (%)		*P*^1^
	Baseline (*n* = 47)	Follow-Up (*n* = 47)	
Age (yr)	15.3 ± 1.44	16.2 ± 1.41	**0.001**
Height (cm)	166.7 ± 8.51	167.3 ± 8.79	0.731
Weight (kg)	61.3 ± 17.01	63.7 ± 16.05	0.493
BMI	21.8 ± 4.67	22.6 ± 4.65	0.465
School Type^2^			0.461
*Gymnasium*	20 (42.5)	14 (31.1)	
*Realschule*	19 (40.4)	17 (37.8)	
*Hauptschule*	1 (2.1)	2 (4.4)	
*Other*	7 (14.9)	12 (26.7)	
ICD-10 Diagnoses			0.372
*F0.X*	0 (0.0)	0 (0.0)	
*F1.X*	9 (19.1)	15 (31.9)	
*F2.X*	0 (0.0)	0 (0.0)	
*F3.X*	30 (63.8)	25 (53.2)	
*F4.X*	18 (38.3)	18 (38.3)	
*F5.X*	8 (17.0)	2 (4.3)	
*F6.X*	20 (42.6)	18 (38.3)	
*F7.X*	0 (0.0)	0 (0.0)	
*F8.X*	0 (0.0)	0 (0.0)	
*F9.X*	10 (21.3)	9 (19.1)	
NSSI frequency (6 months)	33.9 ± 38.60	16.1 ± 30.31	**0.017**
DIKJ	31.0 ± 7.44	21.6 ± 12.28	**<0.001**
SCL-90	1.74 ± 0.74	1.25 ± 0.81	**0.005**
BPD-Criteria	3.36 ± 2.15	2.95 ± 2.29	0.382

*BMI* body mass index, *DIKJ* Depressionsinventar für Kinder und Jugendliche, *SCL-90* Symptom-Checklist-90, *BPD* Borderline Personality Disorder.

^1^Significance: Values refer to differences between groups, with t-tests between the patient groups and Fisher’s exact test for categorical variables.

^2^Hauptschule: secondary school terminating with a lower secondary-school level II certificate: Realschule: secondary school terminating with a secondary-school level I certificate; Gymnasium: secondary school terminating with the general qualification for university entry.

### General procedures

The study comprised three appointments. Diagnostic assessments were conducted during the first, while the second focused on biological assessments, beginning at 8 a.m. with height, weight, and questions concerning nicotine use, recent illnesses, and medications etc. Participants confirmed fasting status, last meal, and cigarette consumption to avoid interference with the blood draw. Before blood collection, participants rested in a supine position for at least 10 min, during which a resting electrocardiogram (ECG) was obtained. Fasting blood was then drawn from the arm by trained personnel, ensuring standardized pre-assessment conditions to prevent exercise-induced increases in AEA concentrations. The third appointment, conducted one year later, mirrored the procedures of the second appointment as a follow up. The treatment regime followed procedures implemented in AtR!Sk [36]. In brief, AtR!Sk adheres to a four step clinical protocol: step 1 (screening) and 2 (diagnostics), comprising the detailed characterization of patients, as described below. In step 3 (treatment) patients are offered different treatment procedures, following a stepped care procedure, including regular visits with specialized doctors, psychotherapy sessions (evidence based short-term CBT, DBT-A), social worker support or (in case of emergency or crisis) inpatient treatment (step 4). The frequency of outpatient (sessions) and inpatient (days hospitalization) treatment was quantified for analyses.

### Measures

Sociodemographic data were collected via a semi-structured interview. NSSI, suicidal thoughts, and attempts were assessed using single items from the German version of the *Self-Injurious Thoughts and Behaviors Interview* (SITBI-G) [40], modified to align with DSM-5 criteria for NSSI. BPD symptoms were evaluated using the relevant section of the *German Structured Clinical Interview for DSM-IV Axis II Disorders* (SCID-II) [41]. Depressive symptoms were measured with the *Depression Inventory for Children and Adolescents* (DIKJ) [42] while overall psychological symptoms were assessed with the *Symptom-Checklist-90*. Childhood maltreatment was evaluated using the *German Childhood Experience of Care and Abuse Questionnaire* [43], adapted from its interview version to include modules on parental care (antipathy/neglect), physical abuse, and sexual abuse. Self-reported cannabis use in the past year was categorized as never, regular, or heavy.

### Sampling procedures

Venous blood was collected between 8:30 a.m. and 9:00 a.m. for endocannabinoid analysis. Blood was drawn into 2.7 ml EDTA tubes, centrifuged at 2000 g for 10 min at 18 °C, and plasma was aliquoted and immediately frozen at −80 °C until analysis. The time to centrifugation was recorded, as AEA release from blood cells is time- and temperature-dependent [44]. Levels of AEA and 2-AG and related endocannabinoid system ligands palmitoyl ethanolamide (PEA) and oleoyl ethanolamide (OEA) in plasma were measured using Liquid Chromatography coupled with Electrospray Ionization Tandem Mass Spectrometry (LC-ESI-MS/MS) following established protocols [45, 46] with detailed analysis procedures provided in the *Supplementary Material*. Due to rapid ex vivo isomerization of 2-AG during extraction and the physiologically negligible amounts of 1-AG, 2-AG and 1-AG peaks were combined to represent total 2-AG levels as previously described [47, 48] and the sum of 2-AG/1-AG compounds is referred to as 2-AG throughout the paper. Data of two participants with an unusually high 2-AG concentration (672.33 and 80.85 nmol/L) were excluded in 2-AG analysis to ensure data integrity, as such extreme values have not been reported and likely indicate error.

### Statistical analysis

Linear mixed-effect models with robust variance estimation were used to address changes in endocannabinoids (AEA, 2-AG, PEA and OEA) and clinical variables (DIKJ, SCL, self-injury in the past 6 months) over time, with the subject ID as random effect. Models on endocannabinoids were adjusted for processing time, BMI and marijuana consumption. In a second step, all models predicting endocannabinoids and clinical symptoms over time, were adjusted for the number of outpatient therapy sessions and days of inpatient hospitalization. For the analyses of the development of BPD symptoms over time, multilevel mixed-effects generalized linear models for binomial data were used and adjusted in line with other clinical variables of interest. To address associations between changes in endocannabinoids and clinical variables, change scores for each variable were created. Their association was assessed using pairwise correlations (zero-order). Further, partial correlations adjusting for processing time, BMI, cannabis consumption, as well as number of outpatient therapy sessions and days of inpatient clinical care, were calculated. To address the predictive value of endocannabinoids at baseline on clinical symptoms over time, linear mixed models (DIKJ, SCL, self-injury past 6 months) and multilevel mixed-effects generalized linear models for binomial data (BPD) were repeated, including baseline endocannabinoid (AEA, 2-AG, PEA and OEA) as predictor, while adjusting for processing time, BMI, and cannabis consumption. All statistical analyses were performed using Stata/SE (Version 18.0; Stata Corp LLC, College Station, TX, USA).

## Results

**Fig. 1:**
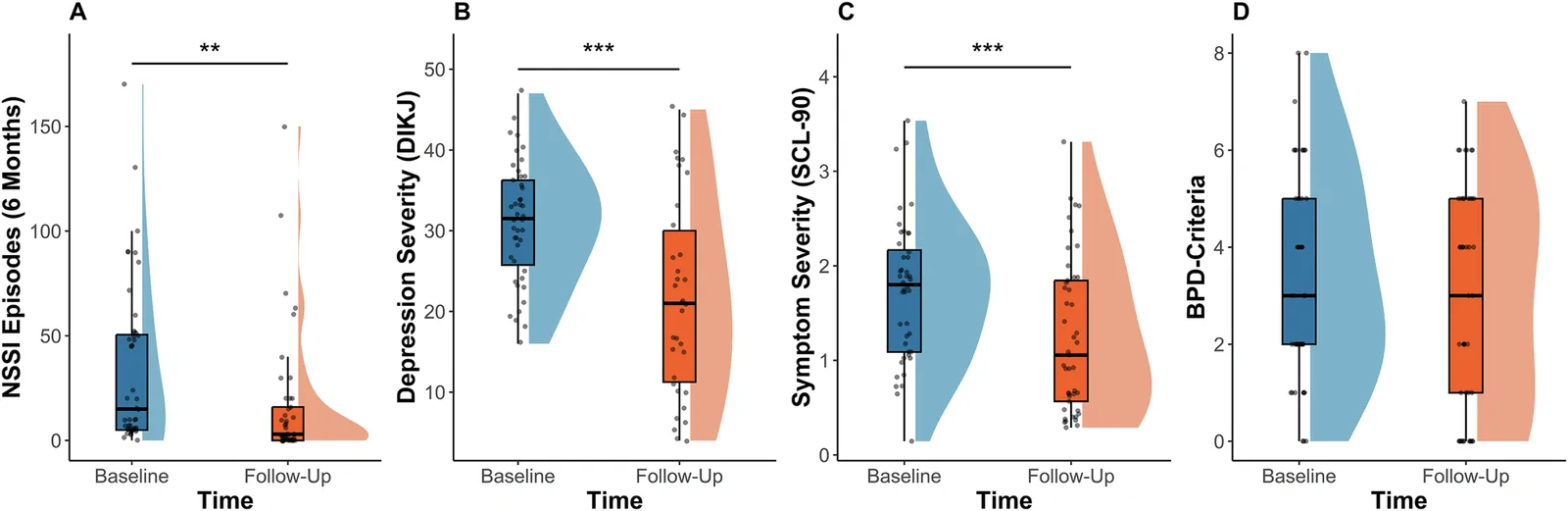
Clinical Characteristics at Baseline and Follow up. After one year, patients showed fewer episodes of non-suicidal self-injury in the past six months (**A**) a significant reduction in depression severity as measured by the DIKJ (**B**), as well as a decrease in global symptom severity (SCL-90) (**C**).The number of borderline personality disorder criteria fulfilled remained unchanged between baseline and follow-up (**D**). (** < 0.01, *** < 0.001).

Complete follow-up data was available for *n* = 47 patients. Sociodemographic and clinical characteristics by time of assessment are provided in Table 1. All participants were of European origin. At baseline, 35 patients (74.47%) reported no cannabis use during the past year, 10 (21.28%) reported moderate cannabis use, and 2 (4.26%) reported heavy cannabis use. Cannabis use did not change significantly over time (Fisher’s exact test, *p* = 0.721). Between assessments one year apart, participants received on average 15.87 outpatient psychotherapy sessions (95% CI [11.08, 20.66]) and 17 days of inpatient clinical care (95% CI [7.92, 26.08]). Patients showed significant improvement with respect to a decrease in the frequency of NSSI (past 6 months; χ^2^_(1)_ = 8.56, *p* < *0.01;* coef = −17.46; 95%CI [−29.17; −5.76]), decrease in depressive symptoms (χ^2^_(1)_ = 22.13, *p* < *0.0001;* coef = −9.66; 95%CI [−13.69; −5.64]) and a decrease in SCL global symptom severity (χ^2^_(1)_ = 21.33, *p* < *0.0001;* coef = −0.48; 95%CI [−0.69; −0.28]). The number of BPD criteria fulfilled, showed no significant change over time (χ^2^_(1)_ = 1.17, *p* = *0.191;* coef = −0.21; 95%CI [−0.52; 0.10]). Findings are illustrated in Fig. 1.

**Fig. 2:**
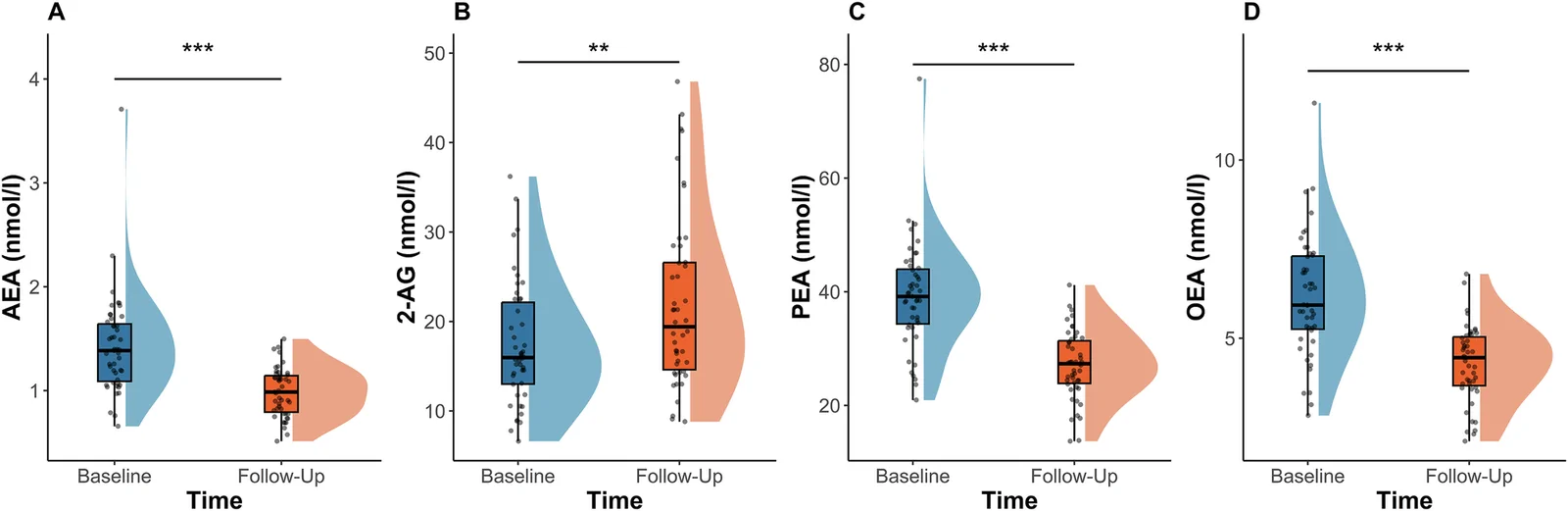
Endocannabinoids at Baseline and Follow up. AEA, PEA and OEA were significantly lower at the follow up appointment (**A, C, D**). 2-AG was significantly higher at follow-up (**B**). Statistics of analysis adjusted for processing time, BMI, and cannabis consumption reported; *** < 0.001.

Endocannabinoids showed significant change over time, indicated by a decrease in AEA (χ^2^_(1)_ = 59.66, *p* < *0.0001;* coef = −0.44; 95%CI [−0.57; −0.31]), decrease in PEA (χ^2^_(1)_ = 64.91, *p* < *0.0001;* coef = −12.25; 95%CI [−15.28; −9.21]), and decrease in OEA (χ^2^_(1)_ = 58.40, *p* < *0.0001;* coef = −1.93; 95%CI [−2.43; −1.42]). 2-AG showed a significant increase over time (χ^2^_(1)_ = 27.10, *p* = *0.003;* coef = 4.52; 95%CI [1.49; 7.54]). Models were robust in unadjusted analyses and analyses adjusted for blood processing time, BMI, and cannabis consumption (data from adjusted models reported). Findings are illustrated in Fig. 2.

**Fig. 3:**
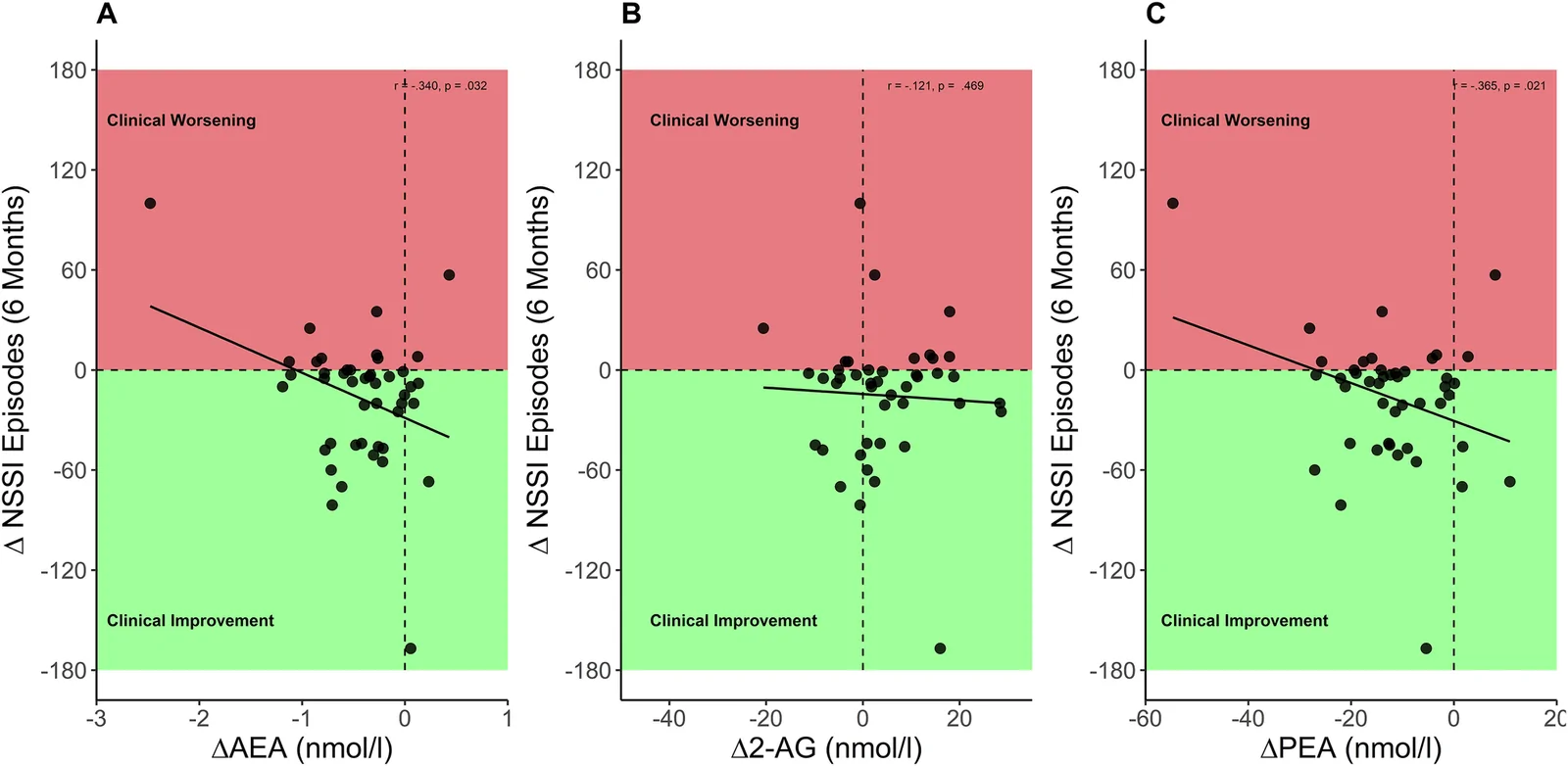
Association between changes in endocannabinoids and improvement in the frequency of non-suicidal self-injury. A decrease in AEA was significantly associated with an increase in NSSI frequency (**A**) and the same was observed for PEA (**C**). There was no association between changes in NSSI frequency and changes in 2-AG (**B**) or OEA (data not shown). Statistics shown are partial correlations adjusted for mean processing time, BMI, cannabis consumption, number of outpatient treatment sessions, and days of inpatient clinical care.

Addressing associations in changes of endocannabinoids and clinical outcomes, change scores (delta approach) illustrated significant association between the relative change in NSSI frequency with the change in AEA (r_(45)_ = -0.329, *p* = *0.027*) and PEA (r_(45)_ = -0.318, *p* = *0.003*). Further, the change in AEA was trend wise associated with the change in SCL global symptom severity (r_(43)_ = -0.304, *p* = *0.053*). No other significant associations between the change in endocannabinoids and clinical outcomes were observed. Zero-order correlations on the change in NSSI frequency showed robust in partial correlations adjusting for mean blood processing time, BMI, and cannabis consumption, as well as intensity of treatment (number of outpatient treatment sessions and days of inpatient clinical care; AEA: r_(45)_ = -0.340, *p* = *0.032;* PEA: r_(45)_ = -0.365, *p* = *0.021*). The association between the change in SCL global symptom severity and AEA showed no robust relationship in the adjusted analysis (r_(45)_ = -0.269, *p* = *0.107)*. Results suggest that a decrease in AEA or PEA was associated with an increase in NSSI frequency. However, these results might be compromised by two participants with either strong improvement or worsening of symptoms (delta self-harm in the past 6 months >100). Removing respective cases, the significant association was no longer detectable. Importantly, the removal of these outliers did not affect the significant differences between baseline and follow-up levels. Nevertheless, the association between symptom trajectories and changes in endocannabinoids appears to be largely driven by these participants, which limits the strength and robustness of this relationship. Findings on NSSI (within the full sample) are illustrated in Fig. 3. Subsequent analyses illustrated that the change in NSSI frequency, was predicted by baseline AEA (χ^2^_(1)_ = 20.74, *p* < *0.001;* coef = 37.15; 95%CI [2.99; 71.30]) but not PEA (χ^2^_(1)_ = 18.50, *p* < *0.01;* coef = 0.12; 95%CI [−1.31; 1.56]) or 2-AG (χ^2^_(1)_ = 18.92, *p* < *0.01;* coef = 0.74; 95%CI [−2.70; 1.75]). Results suggest that adolescents with higher AEA at baseline were more likely to show deterioration of NSSI. However, this analysis included participants with extreme changes in clinical symptoms, and the observed association appears to be driven largely by these cases. After excluding these participants, there was no evidence of an association. Baseline PEA, however, predicted the change in BPD criteria (χ^2^_(1)_ = 14.95, *p* = *0.011;* coef = 0.06; 95%CI [0.01; 0.11]).

## Discussion

This study is the first to longitudinally investigate within-subject changes in endocannabinoids in adolescents engaging in NSSI. Our previous findings showed reduced AEA levels in adolescents with NSSI, associated with the severity of symptoms [35]. We hypothesized correspondingly that AEA levels would increase with successful treatment over time. However, contrary to this hypothesis, the present findings show that AEA levels were lower at the follow-up appointment. Interestingly, 2-AG was higher after one year. According to our baseline findings but not matching the overall trends by timepoint, a decrease of AEA levels was associated with a smaller reduction in NSSI frequency. However, this needs to be interpreted with caution because of two participants with extreme changes in clinical symptoms that might drive the respective association.

### Endocannabinoids in NSSI,BPD and associated co-morbidities

NSSI is a transdiagnostic phenomenon, and alterations in endocannabinoid functioning have been linked to several psychiatric disorders that show high comorbidity with NSSI [33, 49, 50] and are also represented in our sample. BPD is particularly relevant in this context, as NSSI constitutes one of its diagnostic criteria. In our sample, BPD criteria remained stable over time, and in a previous study using a transdiagnostic adolescent sample we did not observe associations between BPD criteria and plasma endocannabinoid levels [35]. To our knowledge, no studies have specifically examined endocannabinoids in individuals with NSSI outside of BPD samples. Although findings from BPD samples cannot be directly compared with those from a transdiagnostic NSSI sample, they represent the closest available literature for contextualizing our results. Some similarities and discrepancies across studies may therefore reflect these differences in sample characteristics. Reduced AEA levels in adolescents with NSSI are consistent with elevated FAAH binding in the PFC and amygdala of patients with BPD and a positive association between FAAH binding and impulsivity, as demonstrated in two PET imaging studies [51, 52]. However, findings contradict those on baseline AEA blood levels in BPD patients [53, 54], which are attributable to several factors, including the presence of comorbid diagnoses, the use of psychotropic medications, and methodological differences in sample matrices (e.g., serum, plasma, or hair). In line with our findings, reduced hair AEA levels were found in patients with BPD [55]. Blood-derived endocannabinoids, including plasma AEA, are influenced by acute stress and external factors such as physical activity [56], feeding behavior [57, 58], and cannabis use [59]. Although we accounted for these factors in our analysis, they should be considered when interpreting the results, as they may contribute to the heterogeneity across studies, in particular in contrast to studies in adult BPD patients.

### Biomarkers in context: baseline and adaptability

The present findings of endocannabinoid system alterations in NSSI are consistent with a growing body of literature across diverse psychiatric disorders in both adult and pediatric cohorts. Although circulating endocannabinoid levels are frequently correlated with symptom severity, the polarity of these associations might be disorder-specific. It is important to note that NSSI is a transdiagnostic behavior, and in this sample a substantial proportion of participants also met diagnostic criteria for MDD or anxiety disorders. The endocannabinoid system may function differently across these conditions, making it difficult to attribute the observed endocannabinoid alterations specifically to NSSI. Larger samples will be needed to allow stratified analyses by comorbid diagnoses to better disentangle these relationships. One might speculate that pathophysiological relevance lies not in the absolute magnitude or direction of endocannabinoid dysregulation, but rather in the perturbation of homeostatic set points and, critically, in an impaired capacity for dynamic allostatic adaptation within the endocannabinoid system. Consequently, deficits in system flexibility and regulatory responsiveness, irrespective of whether endocannabinoid tone is elevated or diminished, may constitute a core mechanism contributing to symptom expression and treatment resistance. For example, Marusak et al., studying a pediatric sample with anxiety disorders, observed that higher baseline AEA and lower baseline 2-AG was associated with greater symptom severity, whereas lower baseline AEA and higher 2-AG predicted a more favorable treatment response [60]. In adolescents with NSSI, lower baseline AEA was associated with greater symptom severity [35], and higher baseline AEA predicted poorer treatment response. However, this latter association was not robust and became non-significant after excluding two participants who showed particularly large changes in symptoms over the year.

### Role of neurodevelopment and the endocannabinoid system

It is important to emphasize that these observations may be specific to pediatric populations, particularly during adolescence, a developmental window marked by significant and dynamic modulation of circulating endocannabinoid signaling pathways (for an extensive review of the endocannabinoid system in neurodevelopment, see [61]). Preclinical studies suggest that CB1 receptor expression in the prefrontal cortex and limbic regions peaks during early adolescence and subsequently declines into adulthood [61–63]. Interestingly, this developmental trajectory parallels the epidemiology of NSSI, which typically emerges around age 14 and declines into adulthood. At the same time, translating findings from animal models to humans requires caution. A recent cross-species study demonstrated substantial differences in peripheral eCB expression patterns across mice, rats, and nonhuman primates [64], highlighting potential limitations in generalizing preclinical findings to humans. To date, only one study has examined circulating endocannabinoid levels across the human lifespan including adolescents [38]. In this serum- endocannabinoid based study with seven age groups, the closest comparable groups for this sample were 5–15 years and 15–30 years. In group comparisons spanning ages 5–30 years, serum AEA and 2-AG levels appear visually stable in females, providing no clear indication that the changes in circulating endocannabinoids observed in the present longitudinal sample are merely attributable to age. However, the broad age range in this study complicates interpretation. Recent longitudinal research examining hair endocannabinoids has demonstrated substantial variability in youth [65, 66]. Consequently, current human evidence regarding normative developmental changes in circulating endocannabinoids during adolescence remains limited, underscoring the need for longitudinal studies in well-characterized adolescent samples.

Among adults, hair AEA levels exhibited a negative correlation with depressive symptoms one year later, which was similarly inverse at the subsequent time point [67]. It is noteworthy that in adolescents, only the negative correlation between hair cortisol and depressive symptoms, consistent with findings in adults, was observed [68]. Children and adolescents with depression exhibited lower hair AEA levels compared to healthy control participants, yet no longitudinal association between AEA levels and depressive symptoms was identified [68]. Our results align with and extend these findings, showing a decrease in plasma AEA concentrations in adolescent NSSI patients over time. A recent study in adults examined longitudinal changes in endocannabinoids measured in hair and symptom trajectories following a multimodal, trauma-focused inpatient treatment. At baseline, hair AEA levels were negatively associated with depressive and anxiety symptoms, but not with PTSD symptoms, similar to our findings of a negative association between plasma AEA and NSSI frequency. However, in this adult sample, changes in symptom severity over the course of treatment were not related to changes in hair endocannabinoid levels. The discrepancy observed between the findings in adults and adolescents may indicate that the neurobiological mechanisms are influenced by factors specific to developmental status. Additionally, the duration of stress exposure may drive adaptive changes in stress response systems over time. Given the challenges in pinpointing the onset of psychopathology and the observation that most psychiatric disorders emerge during adolescence [69], it is reasonable to postulate that the age of the individual often serves as a surrogate marker for the duration of stress exposure and this might also explain differences in results in adults and pediatric psychiatric samples and the endocannabinoid system. This is particularly relevant, as alterations in CB1 receptor availability have been observed in both preclinical and human studies of trauma-related disorders [70], yet the findings are not fully consistent, underscoring the need for translational research to clarify these associations. Matrix effects may contribute to heterogeneity, as a validation study reported a lack of correlation between hair- and plasma-derived endocannabinoid levels [71].

### Role of pain in NSSI and the endocannabinoid system

Comparing our sample with other pediatric psychiatric endocannabinoid studies mainly looking at anxiety disorders [60] or depression [72], a key distinction in our sample of adolescents with NSSI and adverse childhood experiences is the repetitive experience of painful sensations by means of self-injury. The endocannabinoid system plays a crucial role in pain modulation, influencing both the sensory and affective dimensions of pain [73]. While, preclinical studies suggest an analgesic function of endocannabinoids [73, 74], evidence in humans is lacking and one recent study in humans found no association between quantitative sensory testing and serum endocannabinoids [75]. One might speculate that reduced endocannabinoid levels following decreased NSSI reflect diminished molecular peripheral pain responses. In summary, our findings on circulating endocannabinoids in adolescents with NSSI and adverse childhood experiences should be considered in the context of neurodevelopmental status and the characteristic features of NSSI, including initially lower AEA levels and repetitive experience of painful sensations.

### Linking stress system activation to symptom change

Alterations in neurobiological systems involved in stress responsivity have previously been reported in patients with NSSI, primarily within the HPA-axis rather than the endocannabinoid system. For example, individuals with NSSI have been shown to exhibit a blunted cortisol response to stressors [76]. Similar to the literature on basal functioning of the endocannabinoid system, findings regarding basal HPA-axis functioning in NSSI are heterogeneous. Some studies have reported a greater cortisol awakening response [77] and elevated hair cortisol concentrations [78] others found lower serum cortisol levels in individuals with NSSI [79]. Importantly, unstimulated cortisol was not associated with the course of NSSI over time [80]. One interpretation that may also be relevant to alterations in the endocannabinoid system is that individuals with NSSI are often exposed to chronic stress, and prolonged stress exposure may lead to adaptations in neurobiological stress-response systems such as the HPA axis and the endocannabinoid system. Accordingly, alterations in basal peripheral markers may be difficult to detect consistently, whereas stress-induced changes and system reactivity may be more strongly affected and therefore represent more sensitive indicators of dysfunction than resting baseline levels.

The heterogeneity observed in baseline and stress-induced cortisol findings may provide useful context for interpreting endocannabinoid alterations in NSSI. In healthy participants AEA increased following social stress induced by the TSST [81]. However, Spohrs et al. found that AEA increased after a fear extinction paradigm in healthy volunteers [82]. Assessing stress-induced changes in endocannabinoids in patients with NSSI may therefore provide important insights into the functional role of this system. Moreover, given that acute stress can directly influence circulating endocannabinoid levels, it is critical to consider stress levels at the time of neurobiological assessment when interpreting longitudinal findings. Changes in endocannabinoid levels may reflect transient effects of acute stress (or its absence) during assessment rather than stable alterations in endocannabinoid system functioning. Future studies should therefore account for subjective acute stress at the time of biological sampling to better differentiate acute stress-related fluctuations from more persistent dysregulation of the endocannabinoid system. The involvement of the HPA axis is particularly relevant because the endocannabinoid system interacts closely with HPA-axis regulation [31, 83]. For instance, activation of corticotropin-releasing hormone receptors has been shown to increase FAAH activity [28]. Although interactions between the HPA axis and the endocannabinoid system are well established at the molecular level and in animal models, human studies have not consistently demonstrated associations between peripheral markers of cortisol and circulating endocannabinoids [84]. Consequently, conclusions regarding an integrated role of the HPA axis and endocannabinoid system in NSSI remain speculative.

If interpreting endocannabinoid level changes as surrogate marker for activation of the stress response systems one reason might be that current evidence-based psychotherapies for NSSI primarily enhance patients’ capacity to manage stressors (e.g., by adopting alternative coping mechanisms other than NSSI). This limitation reflects a broader issue in psychotherapy: while effective at addressing behavioral symptoms, these therapeutic approaches often fail to directly target underlying neurobiological processes. Consequently, reduced AEA levels may continue to indicate prolonged stress exposure and are therefore not a predisposing risk factor for the development of NSSI but rather a consequence of heightened stress, representing a physiological reaction.

Conversely, elevated AEA levels have been associated with positive outcomes in stress regulation: For example, higher AEA concentrations were found to promote the extinction of conditioned fear in a human experiment, attenuate emotional responses to stress and protect against negative emotional consequences of stress, thereby enhancing resilience against stress-related conditions [84, 85]. These findings suggest that while a temporary decrease in AEA is necessary for mounting an effective stress response, prolonged suppression, particularly in the absence of ongoing stress, may prove detrimental. Our findings align with this theoretical framework: We observed that lower baseline AEA levels correlated with greater symptom severity [35], consistent with previous reports of reduced AEA in PTSD [86] and evidence from proof-of-concept studies demonstrating therapeutic potential of FAAH inhibition to elevate AEA and facilitate fear extinction [84]. These findings motivated FAAH inhibitor clinical trials in PTSD; however both phase 2 trials with different FAAH inhibitors failed to achieve primary endpoints (JZP-150: NCT05178316; JNJ-42165279: [87, 88]). Our longitudinal data, documenting endogenous endocannabinoid trajectories without pharmacological intervention, align with these negative clinical trial outcomes. Notably, despite the baseline association suggesting that increased AEA might confer clinical benefit [35] we overall saw lower AEA and lower symptoms of depression and NSSI. Importantly, changes in clinical symptoms were not robustly related to changes in endocannabinoids.

### Endocannabinoid hypothesis of long-term vulnerability in NSSI

Lastly, our data provide a neurobiological framework for understanding why adolescents who engage in NSSI, a behavior that often declines in early adulthood remain at elevated risk for other psychiatric disorders [89]. Notwithstanding the improvement in NSSI symptoms, persistently reduced AEA levels may signify a long-term risk factor, given that alterations to the endocannabinoid system persist post-treatment. These findings are consistent with the hypothesis that elevated AEA levels confer resilience in the development of psychiatric disorders like substance use disorder after childhood trauma [90] and that pharmacological interventions aimed at increasing AEA through FAAH inhibition attenuate stress induced negative affect [84]. However, in our sample, as well as in other NSSI cohorts, comorbidity with disorders such as MDD and anxiety is common. It therefore remains speculative whether the observed long-term outcomes are specifically related to NSSI and the endocannabinoid system, or whether other neurobiological mechanisms associated with these comorbidities mediate vulnerability to the development of additional psychiatric symptoms, even after remission of NSSI.

### Limitations and outlook

This study has several limitations. The most significant are the small sample size and the overlap between NSSI and childhood maltreatment, which makes it difficult to distinguish specific effects of NSSI from those attributable to early trauma. Future studies should examine relevant genotypes, especially of the common FAAH rs324420 polymorphism, alongside endocannabinoid concentrations to determine whether genetic factors explain or mediate the findings. However, because this study employed a within-subject design, the observed changes are unlikely to be attributable to FAAH genetic variation, which remains stable over time. While many studies used blood-derived endocannabinoids, hair-derived endocannabinoids may be less influenced by acute stress effects, making them potentially more suitable as biomarkers of endocannabinoid “tone” and for longitudinal research. Overall, the relationship between peripheral alterations in endocannabinoid levels and central synaptic signaling remains poorly understood. One notable limitation of our study lies in our sole reliance on self-reported cannabis use data with no standardized questionnaire from the past year, without access to recent cannabis use confirmed by an objective measure such as urine toxicology. Our analysis controlled for past-year cannabis; however, this was assessed using a single-item categorical self-report measure. Consequently, potential confounding effects of cannabis use cannot be fully ruled out. Future studies should assess cannabis use with greater granularity and include objective markers for more robust evaluation. Additionally, while the prevalence of substance use disorders is relatively low among children and adolescents compared to adults, future research should account for the use of other substances, particularly alcohol and opioids, due to their established influence on the endocannabinoid system [91, 92]. The observed association between endocannabinoid changes and symptom changes was notably influenced by two patients who exhibited large shifts in clinical symptoms. These patients did not qualify as outliers in either baseline or follow-up data when assessed independently of symptom change. Additionally, since clinical symptom changes are less susceptible to sampling errors compared to biological markers, we chose to retain their data in the analysis. However, to ensure transparency and improve interpretability, we have acknowledged this limitation both in the Results section and here in the Limitations. All participants in our sample were of European origin. It is important to note that heterogeneity in endocannabinoid levels has been observed in ethnically diverse samples [85, 93] and therefore our results are not generalizable to other populations. Finally, as our sample consisted exclusively of female adolescents, future investigations should include individuals of all genders to achieve a more comprehensive understanding of the endocannabinoid system in patients with NSSI. It is imperative to critically evaluate the utility of plasma AEA as a biomarker because of its variability through development and following stress exposure [49]. Insightful information might come from further investigating FAAH activity, either by PET imaging, genotyping the FAAH-SNP as a genetic risk biomarker or by measuring FAAH activity in the blood, a novel strategy recently demonstrated in individuals with chronic non-medical prescription opioid use [94]. This is particularly relevant for children and adolescents, where the endocannabinoid system appears to exhibit considerable variability in response to stress.

### Conclusion

Our findings contribute to the growing evidence that the endocannabinoid system is altered in adolescents with NSSI. Interestingly, AEA levels decreased over time and there were higher 2-AG levels detectable after one year. However, the changes in 2-AG were not associated with symptom changes and the changes in AEA were surprisingly negatively associated with symptom improvement during treatment but likely driven by 2 patients with extreme changes in clinical presentation. Persistently low AEA levels, however, may elevate the risk of other psychiatric disorders in adulthood, a notable clinical challenge for individuals with a history of adolescent NSSI. Given the role of the endocannabinoid system in mitigating stress-induced negative affect, therapeutic strategies aimed at enhancing endocannabinoid signaling may be more effective for addressing long-term outcomes rather than providing immediate symptom relief in this population.

## Supplementary information

Supplementary Information

## Data Availability

Research data are not shared due to legal and privacy issues.
